# Supplementary material for: Absence of Host-Specific Genes in Canine and Human Staphylococcus pseudintermedius as Inferred from Comparative Genomics
Source: Antibiotics (Basel). 2021 Jul 14;10(7):854. doi: 10.3390/antibiotics10070854 (PMC8300826; doi:10.3390/antibiotics10070854)
Supplement: Supplementary file 1 [file antibiotics-10-00854-s001.zip › Supplementary figure S3_Wegener_MDPI_Antibiotics.pdf]

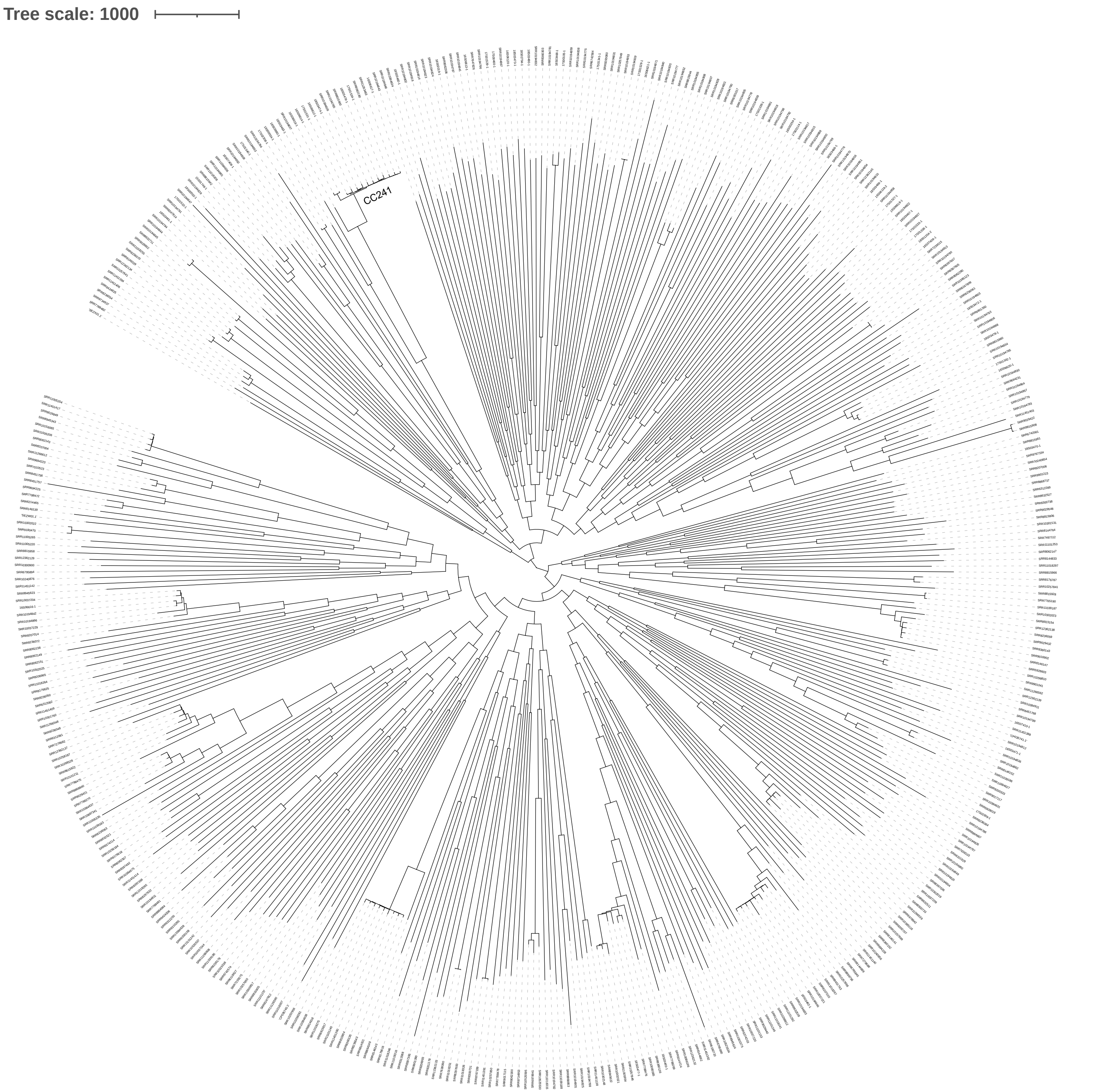

Supplementary figure S3: Phylogenetic tree of MSSP isolates.

This tree compares MSSP isolates from this study with publicly availlable genomes (Tyson *et al.*, 2021; Haenni *et al,*. 2020, Little *et al.*, 2019,)
