# Supplementary figures and images for: Absence of Host-Specific Genes in Canine and Human Staphylococcus pseudintermedius as Inferred from Comparative Genomics
Source: Antibiotics (Basel). 2021 Jul 14;10(7):854. doi: 10.3390/antibiotics10070854 (PMC8300826; doi:10.3390/antibiotics10070854)

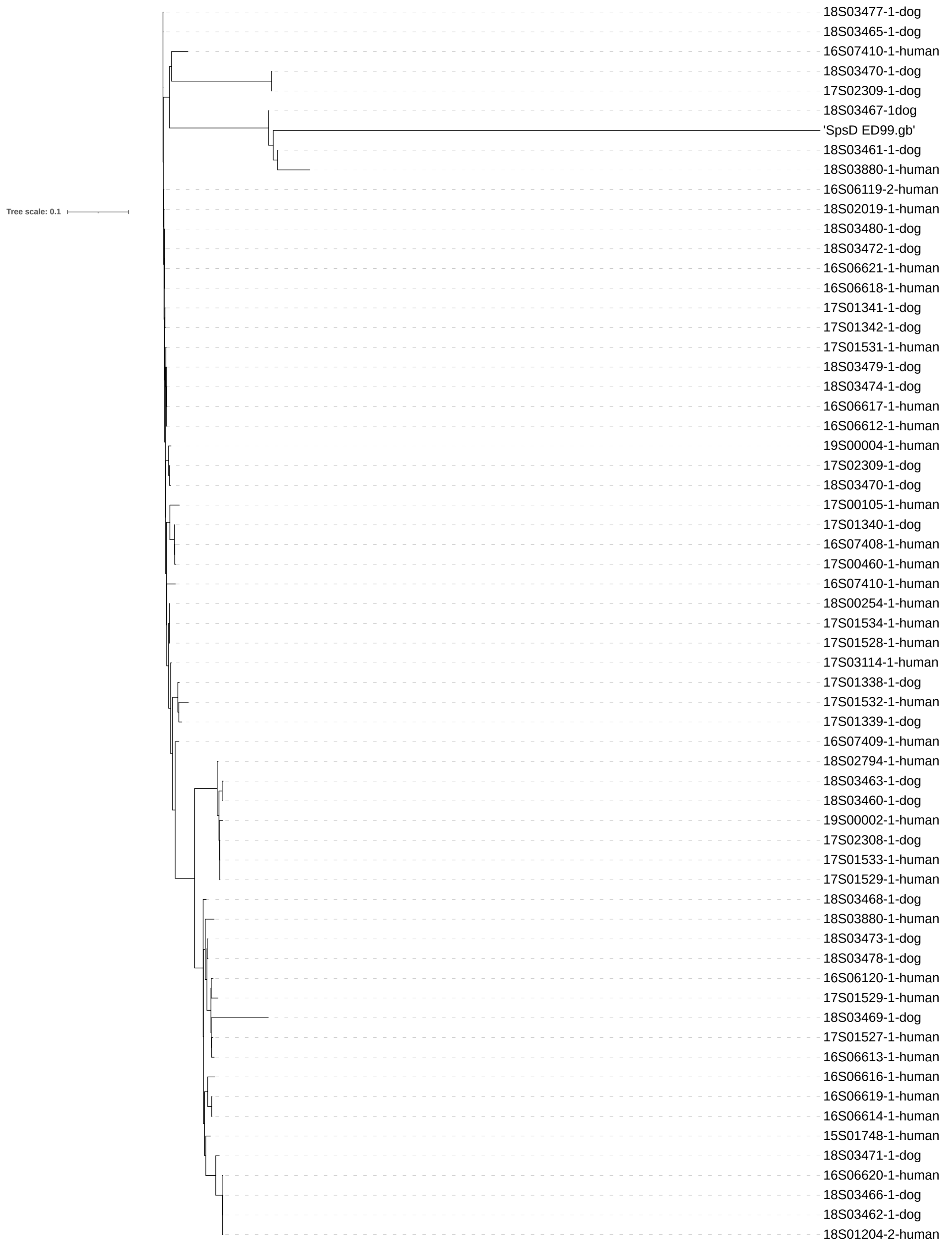

Supplementary Figure S1: Tree of *spsD* genes of dog and humans

Supplement: Supplementary file 1 [file antibiotics-10-00854-s001.zip › Supplementary Figure S1_Wegener_MDPI_Antibiotics.pdf]

Tree scale: 0.1

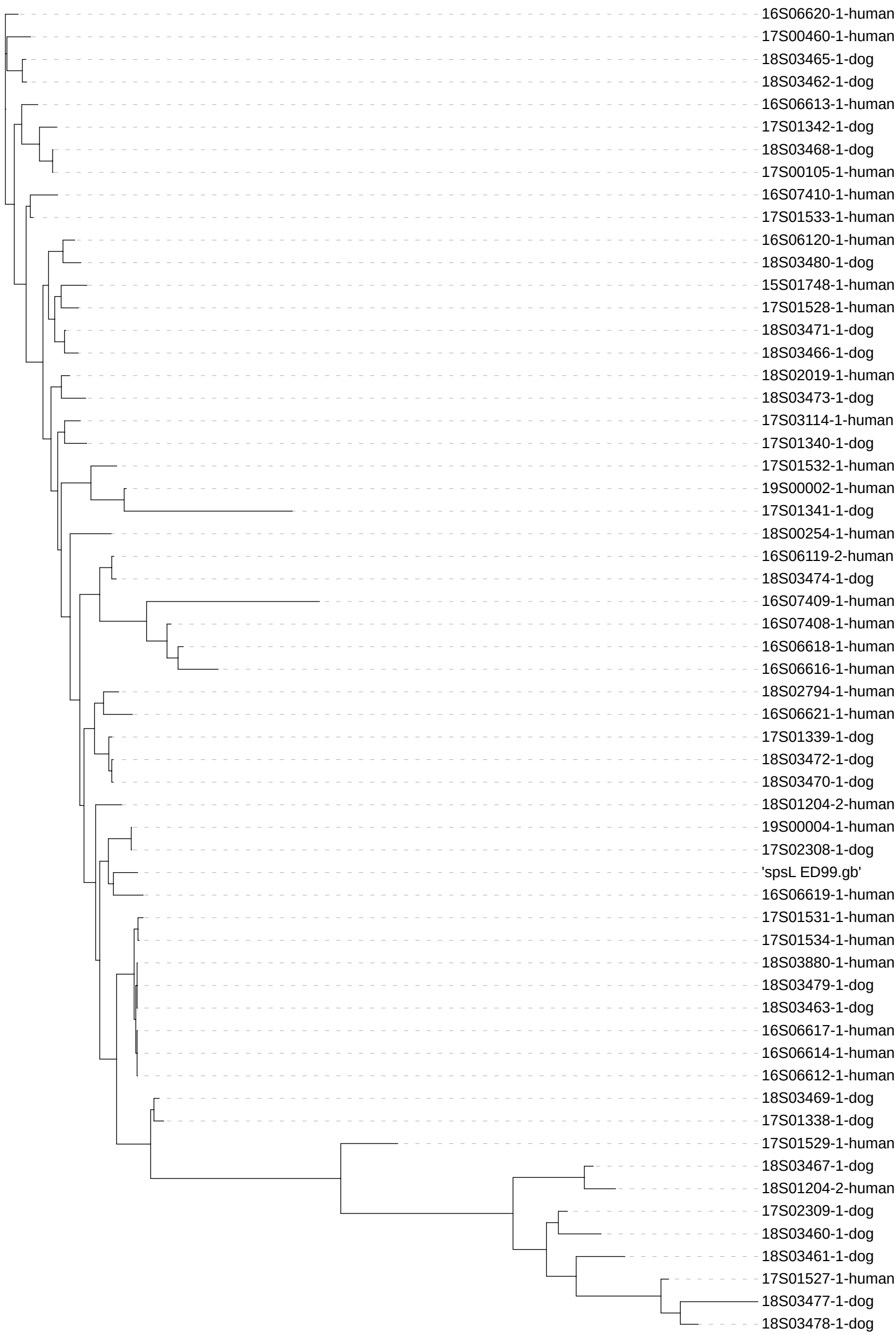

Supplementary Figure S2 : Tree of spsL genes of dog and humans

Supplement: Supplementary file 1 [file antibiotics-10-00854-s001.zip › Supplementary Figure S2_Wegener_MDPI_Antibiotics.pdf]
